# Complete Genome Sequences of Cluster F Mycobacteriophages EleanorGeorge, Mattes, and Spikelee

**DOI:** 10.1128/MRA.00660-19

**Published:** 2019-08-08

**Authors:** Christina Jacob, Yunia Alvarez, Cynthia Castro, Katherine Clarke, Linda Dantico, Bernadette J. Connors

**Affiliations:** a Department of Science, Dominican College of Blauvelt, Orangeburg, New York, USA; DOE Joint Genome Institute

## Abstract

EleanorGeorge, Mattes, and Spikelee are mycobacteriophages isolated from different soil samples using Mycobacterium smegmatis mc^2^155 as the host. Each was obtained using direct isolation techniques, purified, and then sequenced. Based on sequence similarity, all three belong to the F1 subcluster and are temperate phages.

## ANNOUNCEMENT

EleanorGeorge, Mattes, and Spikelee were directly isolated from soil using the actinobacterial host Mycobacterium smegmatis mc^2^155. Two rounds of purification were done, with all methods followed as described in the Howard Hughes Medical Institute (HHMI) Science Education Alliance-Phage Hunters Advancing Genomics and Evolutionary Science (SEA-PHAGES) (1) Discovery Guide (https://seaphagesphagediscoveryguide.helpdocsonline.com/home). The plaques were lightly turbid, indicating that each of these three phages is temperate, capable of integrating the genome into that of its host until the conditions are right for it to transition to the lytic cycle. DNA was purified using the Wizard Promega DNA cleanup kit, and sequencing libraries were prepared from genomic DNA using an Ultra II FS kit with dual-indexed barcoding (New England Biolabs). Libraries were then pooled and run on an Illumina MiSeq instrument, yielding at least 670,000 single-end 150-base reads and at least 1,630-fold coverage for each genome. Assembly was completed using Newbler version 2.9, and for each genome, a single and complete phage contig was produced that was checked for accuracy and phage genomic termini using Consed version 29 (2). The genomes were annotated using DNA Master (3), while GLIMMER (4), GeneMark (5), and Starterator (http://seaphages.org/software/) were used to predict gene starts. ARAGORN and tRNAScan were used to detect tRNA (6, 7). Using predicted sequences derived from autoannotation through DNA Master, function was verified using BLASTp (BLASTDBv5) on the NCBI nr and actinobacteriophage databases (E value of 10E−7 or less), as well as the HHpred database (a probability value of 90% or better was sufficient evidence for function if the query aligned the full length of the subject match or if the alignment included the functional domain) (8–10). Function using synteny was determined using Phamerator (11). Phage genome features are listed in Table 1.

**TABLE 1:** Mycobacteriophage genome details

Phage name	GenBank accession no.	SRA accession no.	Genome length (bp)	GC content (%)	No. of CDS^*a*^	Shotgun coverage (×)
EleanorGeorge	MH669001	SRX6383939	59,482	61.7	108	4,015
Spikelee	MH669014	SRX6383942	58,604	61.0	104	1,629
Mattes	MH155871	SRX6383941	58,074	61.3	105	2,103

a CDS, coding sequences.

The genomic diversity of mycobacteriophages is evidenced by their classification into 26 parent clusters (A to Z) (12), with clusters assigned using nucleotide similarity to other sequenced phage genomes (13). Cluster F has 168 unique phages among five subclusters. F1 contains the largest number of sequenced phages (157 in total). According to the gene content tool on PhagesDB (14), EleanorGeorge and Mattes share 64.8% nucleotide similarity but were only ∼48% similar to Spikelee. According to Phamerator, which classifies predicted proteins into “phamilies” of related sequences and arranges them into genome maps, there is greater similarity in the first half of the genome, with the iconic synteny of the major tail protein, tail assembly chaperone, tape measure protein, and minor tail proteins conserved among all three phages (15). The integrases of Mattes and EleanorGeorge showed significant similarity to one another according to Clustal W Omega multiple sequence alignment (16) (97.85% similarity between Mattes and EleanorGeorge using default settings for protein alignments; 22% similarity of Mattes and EleanorGeorge to Spikelee). Many of the genes in the second half of these genomes cannot be assigned functions, and this region is the source of the greatest variability on genome maps. Some notable exceptions are multiple genes encoding glycosyltransferase, galactosyltransferase, and methylase proteins. Continued exploration of cluster F phage genomes may elucidate additional novel genes in the second half of the genomes.

### Data availability.

The genome sequences reported here have been deposited in GenBank under the accession numbers listed in Table 1 (BioProject number PRJNA488469). The SRA numbers are also provided in Table 1.
